# Atypical Gastric Ulcer in an Elderly Cocaine User

**DOI:** 10.1155/2013/795258

**Published:** 2013-07-30

**Authors:** Vinaya Gaduputi, Hassan Tariq, Ariyo Ihimoyan

**Affiliations:** Department of Medicine, Bronx Lebanon Hospital Center, 1650 Selwyn Avenue, Suite No. 10C, Bronx, NY 10457, USA

## Abstract

Cocaine or Benzoylmethylecgonine is an alkaloid extracted from the leaves of the Erythroxylon plant, which can cause gastrointestinal ischemia from severe arterial vasoconstriction via stimulation of alpha-adrenergic receptors in the gastric and mesenteric arteries. We report this case of a 65-year-old man who presented with a single massive ulcer at the incisura of the stomach as a result of cocaine use. The size and location of this ulcer were atypical and illustrate the potential for serious gastrointestinal manifestations from cocaine use.

## 1. Introduction

Recent estimates suggest that cocaine use is still rampant with almost 1.9% of population of North America indulging in it, the rate being highest in the world [1]. Cocaine is predominantly available in two forms: cocaine hydrochloride salt which is water soluble and can be injected intravenously and a water insoluble cocaine alkaloid, popularly known as crack cocaine [2, 3]. Cocaine has a very short plasma half-life (0.5 to 1.5 hours) but extended tissue half-life of up to 8 hours [2]. Cocaine has multisystem manifestation with well-recognized gastrointestinal manifestations ranging from gastroduodenal ulceration with perforation [4–8] to intestinal infarction with perforation [9]. Cocaine-associated gastroduodenal ulcers are frequently distributed in the greater curvature, prepyloric and pyloric canal regions of the stomach along with the first portion of the duodenum. We intend to report a rare instance of a cocaine-induced giant ulcer at the gastric incisura.

## 2. Case Report

A 65-year-old African American man presented to the hospital with complaints of epigastric abdominal pain and melena of 3-day duration. The patient also reported progressively worsening fatigue over the preceding three months. His medical comorbidities included essential hypertension and gastroesophageal reflux disease. The patient reported no nonsteroidal anti-inflammatory drugs (NSAIDs) use. The patient admitted chronic heavy smoking for almost 50 years and using cocaine until the day before the presentation. Physical examination revealed a hemodynamically stable, cachectic man with minimal epigastric tenderness and melena upon digital rectal examination. 

**Figure 1:**
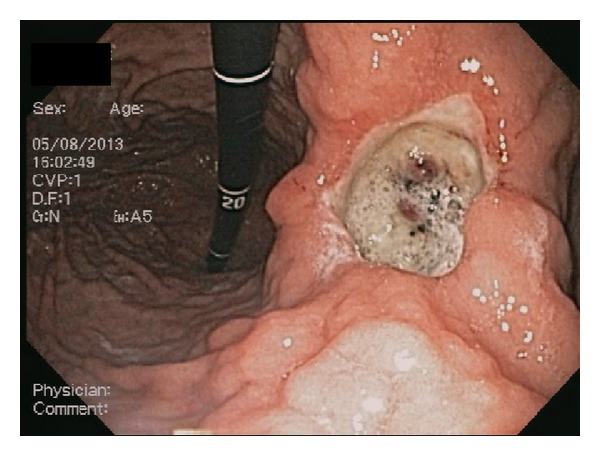
Two visible nonbleeding vessels in the ulcer base.

**Figure 2:**
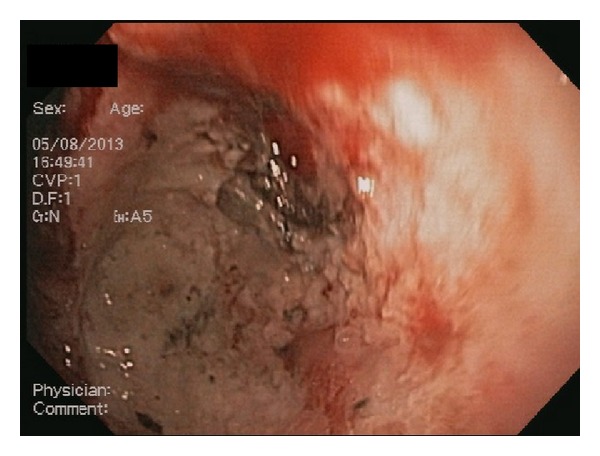
After injection of epinephrine and electrocoagulation with BICAP probe.

Initial set of laboratory studies showed severe anemia (Hemoglobin of 5.6 g/dL). Patient received multiple packed red blood cell (PRBC) transfusions with appropriate improvement in Hemoglobin. He underwent an esophagogastroduodenoscopy (EGD) that revealed a single large 4 cm deep cratered gastric ulcer at the incisura. The base of the ulcer was partly necrotic with eschar formation. There were two visible nonbleeding vessels in the ulcer base (Figure 1). The ulcer site was injected with 1 : 10000 (0.1 mg/mL) epinephrine and subsequently the visible vessels were electrocoagulated with a BICAP probe (bipolar electrocoagulation) (Figure 2). Biopsies were taken from the ulcer site for histology. Biopsies were also taken from gastric antrum and corpus for *Helicobacter pylori* detection, both via rapid urease test (RUT) and histology. Ulcer site histology revealed inflamed granulation type tissue with focal eosinophilic infiltrates. Both histology and RUT were negative for *Helicobacter pylori*. 

Patient ceased to have any further episodes of melena after the therapeutic procedure. Patient was given extensive counseling about the importance of cocaine abstinence and was subsequently discharged.

## 3. Discussion

Multiple mechanisms, by which cocaine can cause gastrointestinal ischemia thereby leading to ulceration and perforation, have been postulated. The most important of these mechanisms is the intense agonist activity of cocaine at the vasoconstrictive alpha-adrenergic receptors in the gastric and mesenteric arteries. Cocaine also has been implicated in direct vasculotoxicity by increasing endothelial permeability to low-density lipoprotein (LDL) causing regulated expression of endothelial adhesion molecules [10] which subsequently leads to leukocyte migration and proliferation of adventitial mast cells [11]. Cocaine may lead to accelerated atherosclerosis via platelet activation and subsequent release of mitogens such as platelet-derived growth factor, epidermal growth factor, and transforming growth factor-beta [11, 12]. Ulcerogenic potential of cocaine is exacerbated by its inhibitory effect on gastric motility. This antimotility effect is a function of both its anticholinergic properties and direct inhibition of medullary centers that regulate gastric motility and vasomotor activity [5]. Delayed gastric emptying in turn leads to dramatically increased acid exposure times, thereby predisposing to ulcer formation. Inhalation or smoking of crack cocaine, as seen in our patient, often leads to higher peak drug levels and much more systemic complications, due to rapid absorption in the extensive pulmonary vascular bed [3, 6, 13]. 

The increased prevalence of cocaine abuse in younger population explains the mean age of presentation being 30 years for cocaine-induced gastroduodenal ulceration. On the contrary, the peptic ulcer disease is prevalent in much older population occurring in ages ranging between 48 and 65 years [4–7]. The age of presentation of our patient is unusual and is amongst the oldest reported in the literature, which might signify changing patterns of cocaine abuse in inner city populations. It is also of note that our patient did not have any antecedent history of peptic ulcer disease which is seen in more than three-quarters of the patients presenting with cocaine-induced gastroduodenal ulceration [4–6]. Biopsy of gastric mucosa in our patient did not reveal coincident *H. pylori* infection which has been noted to increase the likelihood of ulcer formation [14]. The site of ulceration at the incisura is also highly atypical in our patient as review of the literature showed the most common sites to be the first portion of the duodenum, the prepyloric region of the stomach [4–6], the pyloric canal, and the greater curvature of the stomach [5]. Cocaine has been implicated in giant ulcer formation, as seen in our patient, and this risk is increased significantly with concomitant methamphetamine use [15]. The crux of treatment for cocaine-induced ulceration lies in abstinence from cocaine use and subsequent acid suppression therapy with proton-pump inhibitors. For patients presenting with perforation, omental patching using less invasive laparoscopic techniques is the procedure of choice [4, 16]. Acid-reducing operative procedures are not recommended [16]. It has been noted in the literature that physician should be cognizant of the fact that an ulcer perforation in a cocaine abuser can be very subtle without leukocytosis or pneumoperitoneum on abdominal X-ray [4].

We report this case of cocaine-induced gastroduodenal ulceration to highlight its variegated presentation in terms of age of presentation, symptoms, and areas of involvement. A practicing clinician should consider cocaine amongst differential diagnoses for acute gastrointestinal ulceration and should also be aware of its potentially fatal consequences including perforation or massive hemorrhage.
